# Secondary Crystalline Phases Influence on Optical Properties in Off-Stoichiometric Cu_2_S–ZnS–SnS_2_ Thin Films

**DOI:** 10.3390/ma13204624

**Published:** 2020-10-16

**Authors:** Florinel Sava, Ousmane Diagne, Aurelian-Catalin Galca, Iosif-Daniel Simandan, Elena Matei, Mihail Burdusel, Nicu Becherescu, Virginia Becherescu, Claudia Mihai, Alin Velea

**Affiliations:** 1 National Institute of Materials Physics, Atomistilor 405A, 077125 Magurele, Romania; fsava@infim.ro (F.S.); usman.diagn@gmail.com (O.D.); ac_galca@infim.ro (A.-C.G.); simandan@infim.ro (I.-D.S.); elena.matei@infim.ro (E.M.); mihaita_burdusel@yahoo.com (M.B.); claudia.mihai@infim.ro (C.M.); 2 Faculté des Sciences et Techniques, Université Cheikh Anta Diop, Fann Dakar 5005, Senegal; 3 Apel Laser Ltd., Vanatorilor 25, 077135 Mogosoaia, Romania; becherescu@gmail.com (N.B.); virginia.becherescu@gmail.com (V.B.)

**Keywords:** Cu_2_ZnSnS_4_, magnetron sputtering, structural characterization, optical properties

## Abstract

Cu_2_ZnSnS_4_ (CZTS) is an economically and environmentally friendly alternative to other toxic and expensive materials used for photovoltaics, however, the variation in the composition during synthesis is often followed by the occurrence of the secondary binary and ternary crystalline phases. These phases produce changes in the optical absorption edge important in cell efficiency. We explore here the secondary phases that emerge in a combinatorial Cu_2_S–ZnS–SnS_2_ thin films library. Thin films with a composition gradient were prepared by simultaneous magnetron sputtering from three binary chalcogenide targets (Cu_2_S, SnS_2_ and ZnS). Then, the samples were crystallized by sulfurization annealing at 450 °C under argon flow. Their composition was measured by energy dispersive X-ray spectroscopy (EDX), whereas the structural and optical properties were investigated by grazing incidence X-ray diffraction (GIXRD), Raman spectroscopy and optical transmission measurements. As already known, we found that annealing in a sulfur environment is beneficial, increasing the crystallinity of the samples. Raman spectroscopy revealed the presence of CZTS in all the samples from the library. Secondary crystalline phases such as SnS_2_, ZnS and Cu–S are also formed in the samples depending on their proximity to the binary chalcogenide targets. The formation of ZnS or Cu–S strongly correlates with the Zn/Sn and Cu/Zn ratio of the total sample composition. The presence of these phases produces a variation in the bandgap between 1.41 eV and 1.68 eV. This study reveals that as we go further away from CZTS in the composition space, in the quasi-ternary Cu_2_S–ZnS–SnS_2_ diagram, secondary crystalline phases arise and increase in number, whereas the bandgap takes values outside the optimum range for photovoltaic applications.

## 1. Introduction

The world must meet the requirement for clean electricity (without carbon emissions) generation of 30 terawatts by 2050, associated with the expected increase in global energy demand [1]. Solar photovoltaic systems have great potential to address the challenge of future clean electricity supply on a large scale [2]. The quaternary semiconductor Cu_2_ZnSnS_4_ (CZTS) has gained wide attention and has been intensively investigated as a new generation photovoltaic (PV) absorber material. CZTS is theoretically derived from the CuInS_2_ (CIS) structure, where in the formula unit of Cu_2_In_2_S_4_, two trivalent (In) atoms are substituted with one divalent (Zn) atom and one tetravalent (Sn) atom. This isoelectronic substitution produces a material with many properties similar to the initial compound, however, with the added advantage of containing abundant and cheap elements. The Zn content (79 ppm) and the Sn content (2.2 ppm) in the Earth crust are about 1500 and 45 times greater than that of In, respectively. An evaluation of the minimum cost of raw materials for commercialized PV technologies and emerging PV technologies was done by Wadia et al. [3]. The cost for CZTS is much lower than that of other existing PV technologies.

The desirable properties of CZTS include p-type conductivity, a high absorption coefficient (10^4^ cm^−1^, equivalent to 90% of the incident light) and a bandgap of around 1.5 eV (the theoretical optimum value for solar energy conversion [4]). Another advantage of the similarity between CZTS and CIS is that CZTS may be substituted directly into the standard device structure. The potential of CZTS was recognized by Ito and Nakazawa, who prepared synthetic CZTS films using a powder source and atom beam sputtering and demonstrated a photovoltaic effect at the junction between CZTS and cadmium–tin–oxide [5].

The crystalline Cu_2_ZnSnS_4_ has a tetragonal lattice, in fact, a face-centered pseudo-cubic lattice with F-43m (216) space group (“Zinc blende” type structure) where all the atoms (Cu, Zn, Sn and S) are tetrahedrally coordinated. Since the Cu^+^, Zn^2+^ and Sn^4+^ cations must be arranged regularly, the unit cell becomes tetragonal and its crystallographic space group can be either I-42m (121) (similar to the natural mineral “stannite”—a Zn-poor form of Cu_2_(Zn,Fe)SnS_4_), where the metal atom planes on the *c* axis alternate as: Zn–Sn (in a 2D checkerboard array)/Cu (in a cubic array); or I-4 (82) (similar to the natural mineral “kesterite”—a Zn-rich form of Cu_2_(Zn,Fe)SnS_4_), where the metal atom planes on the *c* axis alternate as: Cu–Sn (in a 2D checkerboard array)/Cu–Zn (in a 2D checkerboard array) [6]. Because the Zn-rich mineral has the I-4 space group, it seems reasonable to consider that the most stable phase of Cu_2_ZnSnS_4_ is a “kesterite” type structure. Another reason could be that the “stannite” type structure is more ordered: higher symmetry elements and a “segregation” of the metal atoms in the planes perpendicular on the *c* axis (Cu vs. Zn/Sn).

Therefore, the two crystallographic structures (“stannite” vs. “kesterite”) differ in the ordering of the Cu^+^ and Zn^2+^ cations, but these cations have the same number of electrons (28), meaning that they have equal “atomic X-rays scattering factors”, so the positions or their ordered/disordered distribution [7] and the presence of these cations in the unit cell cannot be easily determined by X-ray diffraction. Other structural techniques such as neutron diffraction [7,8] or Raman scattering are necessary. Another difficulty in the interpretation of XRD data, comes from the fact that the polycrystalline tetragonal (Cu_2_ZnSnS_4_, Cu_3_SnS_4_, Cu_2_SnS_3_) and cubic (ZnS, Cu_0.67_Sn_0.33_S, Cu_0.75_Sn_0.25_S, Cu_0.5_Zn_0.25_Sn_0.25_S) phases have unit cell parameters with very close values.

The formation of polycrystalline phases such as Cu_2_ZnSnS_4_, and/or secondary phases in Cu–Zn–Sn–S thin films is influenced by their elemental composition. Thus, the deviation from stoichiometric CZTS ratios in the thin films can lead to the formation of secondary phases such as binary Cu_2-x_S [9,10], ZnS [11,12] or ternary Cu_4_SnS_4_ [12], Cu_5_Sn_2_S_7_ [12], irrespective of growth techniques [13]. The best performances of such cells are obtained for thin film compositions quite different from the precise Cu_2_ZnSnS_4_ one (stoichiometric CZTS), especially those with copper deficiencies [14]. The effects of the chemical composition variation in the Cu–Zn–Sn–S thin films have started to be studied by combinatorial deposition [15,16,17]. Chemical composition tuning and defect engineering are needed in order to achieve better solar cell performances in Cu–Zn–Sn–S thin films [16].

This study explores the formation of secondary crystalline phases and their effect on the optical properties in off-stoichiometric Cu_2_S–ZnS–SnS_2_ thin films, obtained by magnetron co-sputtering from three binary chalcogenide targets. Moreover, it also provides useful information for the future development of thin-film CZTS-like solar cells: the influence of chemical composition on the structural and optical properties.

## 2. Materials and Methods 

**Figure 1:**
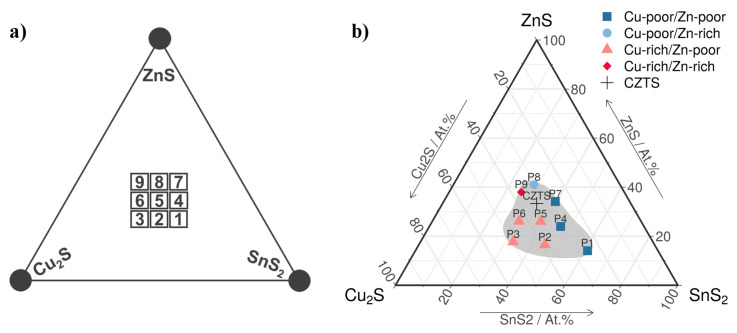
Experimental configuration of the samples and the obtained compositions: (**a**) the positions of the binary targets (circles) and glass substrates (squares) during the magnetron co-sputtering deposition of the Cu_2_S–ZnS–SnS_2_ thin films; and (**b**) the average chemical composition calculated from EDX showed on the Cu_2_S–ZnS–SnS_2_ quasi-ternary diagram. In addition, Cu_2_ZnSnS_4_ (CZTS) is also showed.

The library was synthesized on nine SiO_2_ substrates that were cleaned by successive sonication in different liquids (acetone (Chemical Company, Iasi, Romania), ethanol (Chemical Company, Iasi, Romania), deionized water (prepared using a Thermo Scientific Smart2Putre UV water treatment system, Hatvan Hungary)), dried in a nitrogen flow (Linde, Bucharest Romania) and placed next to each other in the deposition equipment as in Figure 1a. Cu–Zn–Sn–S thin films were deposited by RF magnetron co-sputtering (Gencoa Ltd., Liverpool, UK) from Cu_2_S, ZnS and SnS_2_ binary chalcogenide targets (99.99% purity, Mateck GmbH, Jülich,, Germany, 2 inch in diameter). The magnetron sputtering system is a custom-built setup that consists of a cylindrical deposition chamber (Excel Instruments, Maharashtra, India) with hemispherical up and down caps (Excel Instruments, Maharashtra, India). Three magnetrons are equidistantly placed on the bottom hemisphere. The substrates are placed on a holder in the upper part of the chamber. The distance between the center of the P5 sample and each target was of 11 cm, whereas the rest of the samples were closer to at least one of the targets, resulting in a continuous variation of composition. The angle between the targets and the substrates was 45 degrees. After initially evacuating the chamber at 10^−6^ Torr, Ar gas (Linde, Bucharest Romania) was introduced at a rate of 30 sccm and the pressure inside the chamber, during deposition, was maintained constant at 5 × 10^−3^ Torr. A few minutes of pre-sputtering was performed in order to remove any unwanted contaminants from the target surfaces prior to the deposition process. The sputtering power was set at 80, 50 and 20 W for Cu_2_S, ZnS and SnS_2,_ respectively, leading to a sputtering rate of 0.5 Å/s for each material. The sputtering rates were optimized using an Inficon Q-bridge monitoring software (Bad Ragaz, Switzerland) connected to a quartz microcrystal (Inficon, Bad Ragaz, Switzerland). The deposition time was approximately 35 min, in order to obtain thin films with a thickness of 300 nm. The substrates were not heated during deposition.

Sulfurization annealing was performed by placing the samples in a quartz tube, inserted in a tubular furnace, at 450 °C for 1 h. A continuous flow of 83 sccm Argon was used to transport the sulfur vapors obtained from the evaporation of an upstream sulfur powder. After annealing, the furnace was turned off and the samples were cooled to room temperature in Ar flow in order to avoid oxidation.

The determination of the elemental concentration in the films was carried out by means of energy dispersive X-ray (EDX) spectroscopy using a Zeiss EVO 50 XVP scanning electron microscope (Carl Zeiss, Oberkochen, Germany) equipped with a Bruker Quantax 200 detector (Bruker AXS Microanalysis GmbH, Berlin, Germany).

The investigation of the Cu_2_S–ZnS–SnS_2_ thin films structure was performed by grazing incidence X-ray diffraction (GIXRD) at an incidence angle of 0.3° with a Rigaku SmartLab diffractometer (Rigaku, Tokyo, Japan) provided with Cu K_α_ radiation (λ = 1.54178 Å) and HyPix-3000 2D Hybrid Pixel Array Detector (Rigaku, Tokyo, Japan) (in 0 D mode). The identification of the crystalline phases was performed using the DIFFRAC.SUITE Software package (Bruker, Billerica, USA).

The optical transmission and absorbance spectra were measured using a Variable Angle Spectroscopic Ellipsometer (J.A. Woollam Co., Lincoln, NE, USA), equipped with a high-pressure Xenon discharge lamp (Hamamatsu Photonics K.K., Japan), incorporated in an HS-190 monochromator (J.A. Woollam Co., Lincoln, NE, USA).

Raman spectra were recorded at room temperature, in the 200–450 cm^−1^ range, in backscattering configuration, with a LabRAM HR Evolution spectrometer (Horiba Jobin-Yvon, Palaiseau, France) equipped with a confocal microscope. A He–Ne laser (Horiba Jobin-Yvon, Palaiseau, France) operating at 633 nm was focused using an Olympus 100× objective (Olympus, Tokyo, Japan) on the surface of the samples. Accurate and automated calibration was performed on a standard Si wafer (provided by Horiba Jobin-Yvon, Palaiseau, France) by checking the Rayleigh and Raman signals. The laser excitation power was adjusted to avoid laser-induced heating in the thin films.

## 3. Results and Discussion

### 3.1. Chemical Composition

The average chemical compositions of the Cu_2_S–ZnS–SnS_2_ thin films after sulfurization annealing, obtained from EDX, are shown in Table 1. These values (in the limit of EDX measurement errors) show that the sulfurization does not produce major changes of sulfur content in annealed samples as compared with the as-deposited samples (data not shown). Its role is to avoid sulfur evaporation during annealing, given that sulfur is highly volatile. Starting from the average Cu, Zn and Sn percentages, we calculated the corresponding average percentages of Cu_2_S, ZnS and SnS_2_ in each sample and represented these values in a Cu_2_S–ZnS–SnS_2_ quasi-ternary diagram (Figure 1b). In this diagram, it was easily seen that the average chemical compositions of the P5, P6, P7, P8 and P9 samples were the closest to the stoichiometric CZTS ratio (Cu = 25%, Zn = 12.5%, Sn = 12.5% and S = 50%), the ones of P2, P3 and P4 are at intermediate distance, whereas the one of P1 is further away. We note that the elemental composition of the films varies according to the binary target proximity (Figure 1a). However, during magnetron co-sputtering deposition, some amount of sulfur from the targets no longer reached the glass substrate. Thus, if it was computed, the corresponding average percentage of sulfur ($p_{S}^{ideal}$) necessary to bind with the entire quantity of Cu, Zn and Sn in the sample (to form Cu_2_S, ZnS and SnS_2_), results that for most of the samples, $p_{S}^{ideal}$ is higher than the average percentage of sulfur ($p_{S}$) found by EDX, except for P6, where we have an excess of S. These differences can be seen in the last row of Table 1 ($\frac{p_{S}-p_{S}^{ideal}}{p_{S}^{ideal}}=\frac{\Delta p_{S}}{p_{S}^{ideal}}$). Similarly, the relative deviation from the ideal percentage of each element in the CZTS composition ($\frac{\Delta p_{Cu}}{p_{Cu}^{CZTS}}$, $\frac{\Delta p_{Zn}}{p_{Zn}^{CZTS}}$, $\frac{\Delta p_{Sn}}{p_{Sn}^{CZTS}}$ and $\frac{\Delta p_{S}}{p_{S}^{CZTS}}$) are also shown in Table 1.

In Figure 1b, we can see the labels of the elemental ratios Cu/(Zn+Sn) and Zn/Sn for each sample. If Cu/(Zn+Sn) is greater than 1, the material is considered Cu-rich, otherwise Cu-poor. The same applies for the Zn/Sn ratio, resulting in Zn-poor or Zn-rich compositions. These ratios together with the Cu/Zn ratio, are good indicators of the secondary phases formed after annealing. For instance, a high concentration of copper, when Cu/Zn > 1.5, as in the case of P3, P6, P8 and P9, leads to the formation of secondary Cu–S crystalline phases, whereas a high Zn/Sn ratio is a sign for the formation of ZnS crystalline phases, as seen in P9.

### 3.2. Structural Properties

#### 3.2.1. GIXRD Characterization

**Figure 2:**
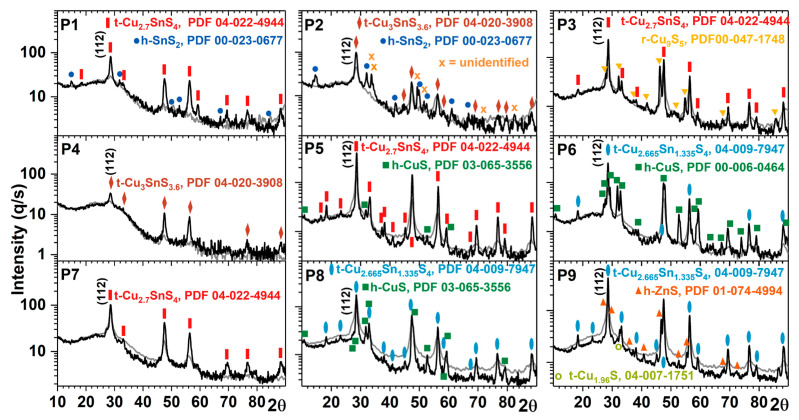
X-ray diffraction patterns of the nine Cu_2_S–ZnS–SnS_2_ thin films in the as-deposited state (gray curves) and after sulfurization annealing at 450 °C for one hour (black curves). The assignment of phases and orientations was done using the International Centre for Diffraction Data (ICDD) database (see Table 3). h = hexagonal; t = tetragonal; r = rhombohedral. The Powder Diffraction File (PDF) for each crystalline phase is also indicated.

The GIXRD diagrams of the thin films in the as-deposited state (Figure 2) show that they are already crystallized to some extent (the percent of initial crystallization *p_i_*, is shown in Table 2). The percent of crystalline phases increases in the samples after the sulfurization annealing (*p_a_* in Table 2), as calculated from the GIXRD diagrams in Figure 2. The percent of crystalline phases before and after annealing (*p_i_* or *p_a_*) was calculated as (*A_C_*/*A_C+Am_*)×100, where *A_C_* is the total area of crystalline peaks (from all the crystalline phases) and *A_C+Am_* is the total area of the peaks (crystalline + amorphous).

It can be observed that, between 14° and 40° in *2θ*, there is a broad peak (centered at 24.57°), which is given, mainly, by the silicate glass substrate. When processing the diffraction curves, to calculate the percent of crystalline phases (*p_i_* and *p_a_*), the signal from the substrate was subtracted.

We observed by analyzing Table 2 and Figure 1b, that the P1 sample (the richest in Sn) is the least crystallized in the as-deposited state, however, it has the highest crystallization rate after annealing. The samples richest in Zn (P8, P9) are the most crystallized in the as-deposited state, and as a consequence they have the lowest crystallization rate after annealing. The same situation was found for the sample richest in Cu (P3).

**Figure 3:**
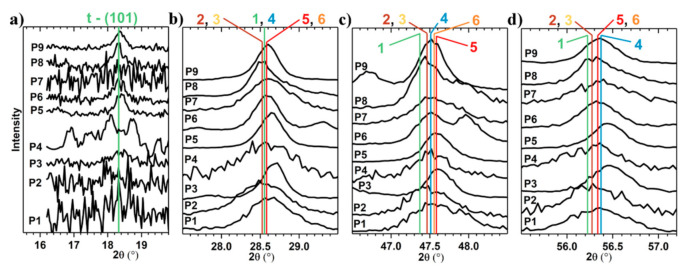
Details of X-ray diffraction patterns of the nine Cu_2_S–ZnS–SnS_2_ thin films. The peaks are normalized (greater fluctuations mean peak’s intensity is lower), so, their widths (*β*), at half of the maximum intensity, can easily be compared. (**a**) The peak at *2θ* ~ 18.32°, was the signature of the tetragonal CZTS phase (the lattice plane: (101)). For the peaks at (**b**) *2θ* ~ 28,54°, (**c**) *2θ* ~ 47.5° and (**d**) *2θ* ~ 56.3°, the vertical lines are the positions where the following phases should be present: 1: Cu_2_ZnSnS_4_, tetragonal, I-42m (121), PDF 00-026-0575; 2: Cu_3_SnS_3.6_, tetragonal, I-42m (121), PDF 04-020-3908; 3: Cu_0.5_Zn_0.25_Sn_0.25_S, cubic, F-43m (216), PDF 04-017-8462; 4: Cu_2.665_Sn_1.335_S_4_, tetragonal, I-42m (121), PDF 04-009-7947; 5: Cu_2.7_SnS_4_, tetragonal, I-42m (121), PDF 04-022-4944; 6: ZnS, cubic, F-43m (216), PDF 00-005-0566.

In Table 3, the majority and the minority phases (italic) and the mean dimension of the crystallites, after the sulfurization annealing of the Cu_2_S–ZnS–SnS_2_ samples, were listed. The percent of the amorphous phase (*p_amorphous_*) was calculated as *p_amorphous_ =* 100 − *p_a_*. For a given sample, the percent of the “X” crystalline phase was given by the ratio *p_a_*×*A_X_/A_C_*, where *A_X_* is the total area of crystalline peaks of phase “X”. Moreover, the position and the half width maximum of the most intense diffraction peaks are compared in Figure 3. Thus, we can observe that in all the samples, the peak position of the majority phase does not match with the Powder Diffraction File (PDF) 00-026-0575 of the CZTS phase. The phases mentioned in Table 3 are those with the smallest deviation from the peaks’ positions found in the International Centre for Diffraction Data (ICDD) database. The most intense peaks (at ~28,54°, ~47.5°, ~56.3°) are very close to the CZTS positions, so it is very probable that CZTS exists as a minority phase (see Raman measurements). The CZTS phase could not be detected by GIXRD because the peaks are broad and a deconvolution was not possible. The majority crystalline phase is most abundantly formed in P5, the sample with the chemical composition closest to ideal CZTS. In contrast to P5, in P8 the majority phase is the least formed (only 45 %), even if its composition is quite close to that of CZTS (Figure 1b). It has the highest percent of crystalline phases (*p_i_*) in the as-deposited state and after annealing this percent remains the same. The average size of the crystallites increases after annealing and has the highest value among all the annealed samples (52 nm, see Table 3). The average size of the crystallites was approximated using the Scherrer equation, *d = Kλ/βcos(θ)*, where *K* is the Scherrer constant, *λ* the wavelength, *θ* the angle and *β* the peak broadening. The instrumental line broadening and *K_α2_* were subtracted a priori.

One should note that the probable presence of macrostrain changes the lattice parameters and shifts the recorded *2θ* values. Thus, the assignment of one phase or another, also considering the small average crystallite sizes, is rather difficult, and the CTS phases can be easily confused with CZTS and vice versa.

#### 3.2.2. Raman Spectroscopy

In the μ-Raman scattering technique, unlike GIXRD, the investigated volume of a sample is very small, allowing a more localized analysis of the thin films. Therefore, the residual phases, revealed by Raman spectra, are not always present in the GIXRD diagrams, because these quantities are below the detection capabilities of XRD.

The Raman peaks for the polycrystalline phases, which are difficult to be discriminated by XRD, are shown in Table 4. There is a strong similarity between the Raman spectra given by “stannite” and “kesterite” type structures and it is difficult to differentiate between them. For “kesterite”, a shift of the most prominent peak (~337 cm^−1^, given only by vibrations of the S atoms) to lower values (between 327 cm^−1^ and 331 cm^−1^) or the existence of a visible shoulder near this peak (at 331 cm^−1^), could indicate the presence of statistically disordered Zn and Cu cations in the “kesterite” structure with copper vacancies, or a stressed “kesterite” phase [18,19].

**Figure 4:**
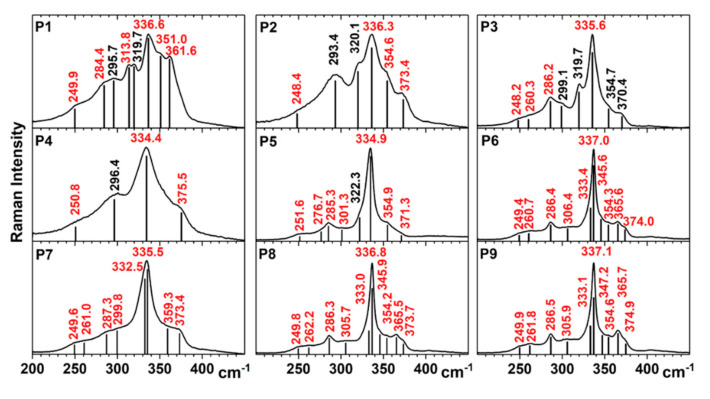
Raman spectra of the combinatorial Cu_2_S–ZnS–SnS_2_ samples after sulfurization annealing. The values of Raman peaks obtained by fitting spectra with Lorentzian curves are indicated.

The Raman spectra measured on the Cu_2_S–ZnS–SnS_2_ samples are shown in Figure 4. The Raman peaks, determined by the fitting of the spectra with Lorentzian curves, can be attributed, according to Table 4, as follows:

- **P1:** CZTS (I-42m) (284.4 cm^−1^, 336.6 cm^−1^, 361.6 cm^−1^), CZTS (I-4) (249.9 cm^−1^, 284.4 cm^−1^, 313.8 cm^−1^, 336.6 cm^−1^, 351.0 cm^−1^), Cu_2_SnS_3_ (I-42m) (295.7 cm^−1^, 336.6 cm^−1^, 351.0 cm^−1^), orthorhombic Cu_3_SnS_4_ (319.7 cm^−1^) [20], hexagonal SnS_2_ (314 cm^−1^, determined by Raman scattering on our SnS_2_ target, data not shown);
- **P2:** CZTS (I-42m) (336.3 cm^−1^), CZTS (I-4) (248.4 cm^−1^, 336.3 cm^−1^, 354.6 cm^−1^, 373.4 cm^−1^), Cu_2_SnS_3_ (I-42m) (293.4 cm^−1^, 336.3 cm^−1^, 354.6 cm^−1^), orthorhombic Cu_3_SnS_4_ (320.1 cm^−1^);
- **P3:** CZTS (I-42m) (286.2, 336.3 cm^−1^), CZTS (I-4) (248.2, 260.3, 286.2, 335.6, 354.7, 370.4 cm^−1^), Cu_2_SnS_3_ (I-42m) (299.1, 335.6, 354.7 cm^−1^), orthorhombic Cu_3_SnS_4_: (319.7 cm^−1^) while for Cu–S the peaks are out of the measured range;
- **P4**: CZTS (I-4) (250.8, 334.4, 375.5 cm^−1^), Cu_2_SnS_3_ (I-42m) (296.4, 334.4 cm^−1^). The most prominent peak in the Raman spectra of P4 is red shifted to 334.4 cm^−1^, and a possible cause could be the smaller size of Cu–Zn–Sn–S crystallites [21] (as was shown by GIXRD measurements);
- **P5**: CZTS (I-4) (251.6, 276.7, 285.3, 301.3, 334.9, 354.9, 371.3 cm^−1^), orthorhombic Cu_3_SnS_4_: (322.3 cm^−1^);
- **P6**: CZTS (I-4) (249.4, 260.7, 286.4, 306.4, 333.4, 337.0, 345.6, 354.3, 365.6, 374.0 cm^−1^), Cu–S out of range;
- **P7:** CZTS (I-4) (249.6, 261.0, 287.3, 299.8, 332.5, 335.5, 359.3, 373.4 cm^−1^);
- **P8:** CZTS (I-4) (249.8, 262.2, 286.3, 305.7, 333.0, 336.8, 345.9, 354.2, 365.5, 373.7 cm^−1^); Cu-S out of range;
- **P9:** CZTS (I-4) (249.9, 261.8, 286.5, 305.9, 333.1, 337.1, 347.2, 354.6, 365.7, 374.9 cm^−1^).

From the Raman spectroscopy results, it can be observed that the CZTS phase is present in all the samples. The formation of the SnS_2_, ZnS and Cu–S binary secondary phases was observed in most samples, except for P4 and P7. The last two samples were Cu-poor/Zn-poor which suggested that a lower quantity of Cu and Zn prevented the formation of a binary secondary phases. P1 is also Cu-poor/Zn-poor, but the high concentration of Sn, due to the proximity to the SnS_2_ target, led to the formation of SnS_2_ secondary crystalline phase.

The Raman spectra revealed that for some samples the Raman peak from ~336 cm^−1^ was very narrow (P3, P5, P6, P8, P9) while for the others it was broad. From Table 3, we saw that the width of this Raman peak was inversely proportional with the average size of the CZTS crystallites.

The full shape of the Raman spectrum can also indicate one compound or another. Compared with the CZTS spectra from the literature [23], P6, P8 and P9 were the closest ones.

### 3.3. Optical Properties

**Figure 5:**
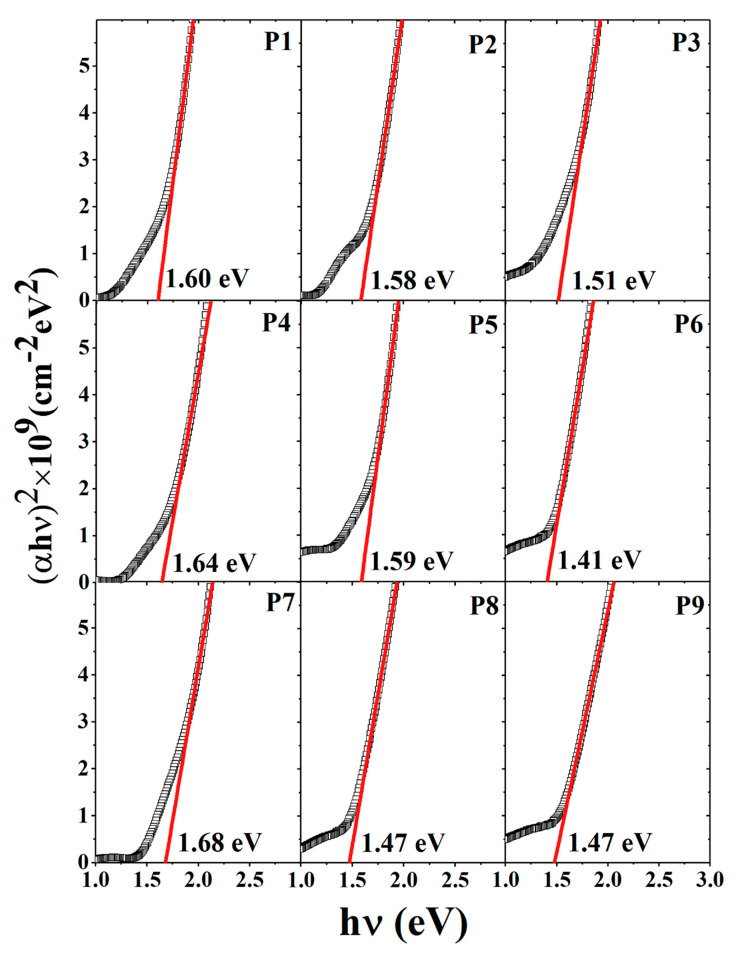
Tauc analysis and bandgap determination for the sulfurization annealed Cu_2_S–SnS_2_–ZnS_2_ thin films.

The optical characteristics of combinatorial Cu_2_S–ZnS–SnS_2_ films were evaluated in terms of optical transmission spectra. From the transmission data, we estimated the bandgap of the films. This estimation was done by extrapolating the linear part of the curve ${\left(\alpha h\nu \right)}^{2}=f\left(h\nu \right)$ to the point ${\left(\alpha h\nu \right)}^{2}=0$ (Tauc analysis). The results are presented in Figure 5.

**Figure 6:**
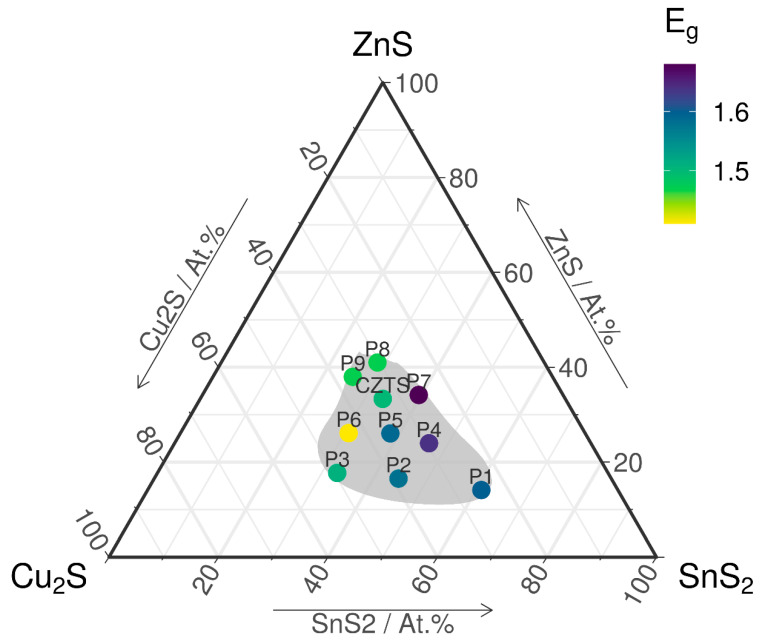
The distribution of the bandgap energy after sulfurization annealing.

The gradient of the bandgap as a function of distance from the CZTS chemical composition in the quasi ternary Cu_2_S–SnS_2_–ZnS_2_ diagram is shown in Figure 6. We can observe that the samples closer in composition to CZTS have similar bandgaps, and as we go further away in the compositional space, the bandgap increases.

The bandgap of the synthesized films varies in the interval of 1.41–1.68 eV. The samples P1, P2, P4 and P7 have bandgaps slightly outside the ideal range (1.4–1.5 eV) of CZTS films [29,30]. Surprisingly, these are the samples with the smallest mean crystallite size. This may be justified by the off-stoichiometry which might favor structure deformation and consequently, modifies the bandgap as proposed by Jeffe and Zunger for chalcopyrite semiconductors [31]. The presence of secondary phases affects the bandgaps of these films [32,33]. The presence of o-Cu_3_SnS_4_ with a bandgap of 1.6 eV [20], h-SnS_2_ which has a bandgap of 1.82 eV, and t-CTS with 1.35 eV [20] are responsible for the variations in the bandgap values. On the other hand, P5 shows a slightly higher bandgap, also obtained by Tanaka et al. [34] in single-phase CZTS films obtained by a sputtering-sulfurization method.

A sulfurization treatment as well as a suitable annealing time are necessary to make improvements in the structural properties and elemental composition [26,33]. As we go further away from CZTS in the composition space, we can observe that the bandgap takes values outside the optimum range for photovoltaic applications.

## 4. Conclusions

Combinatorial Cu_2_S–SnS_2_–ZnS_2_ thin film samples, with a gradient of chemical composition, were synthesized by magnetron co-sputtering on silicate glass substrates using Cu_2_S, SnS_2_ and ZnS binary targets. A ratio of Cu/Zn > 1.5 indicates the formation of secondary Cu–S crystalline phases in P3, P6, P8 and P9, whereas a ratio of Zn/Sn > 1 is a sign for the formation of Zn–S crystalline phases in P9. The XRD diffractograms indicate that annealing in a sulfurized environment increases the polycrystalline fraction of the thin films, but amorphous phases are still present. The sample richest in Sn (P1) is the most amorphous in the as-deposited state, however, it has the highest crystallization rate after annealing, whereas the samples richest in Cu and Zn (P3, P8 and P9) are the most crystallized in the as-deposited state, and they have the lowest crystallization rate after annealing. In all the samples, the majority crystalline phase, obtained by XRD, is a Cu–Sn–S phase that has a stannite type structure, which might be confused with a Cu–Zn–Sn–S phase if a moderate Zn ‘doping’ is present or a macrostrained CZTS can be considered taking into account the presence of Zn inferred from EDX spectroscopy. The CZTS phase could not be detected by XRD also because the peaks are broad and a deconvolution was not possible, but the Raman results show that the CZTS phase was present in each sample. The presence of binary secondary phases (SnS_2_, ZnS and Cu–S) has been observed in most of the samples, except for P4 and P7 (in P5 only 2%). The Raman peak from ~336 cm^−1^ is very narrow for P3, P5, P6, P8, P9, while for the others it is broad, which means that its width is inversely proportional with the average crystallite size of the CZTS phase. The bandgap of all samples lies in the interval of 1.41–1.68 eV. The results show that the probability of having secondary crystalline phases increases as we travel farther away from CZTS and also the bandgap changes outside the optimum range for photovoltaic applications. Finally, this study offers useful insight into how chemical composition influences the structural and optical properties of Cu–Zn–Sn–S films, knowledge which can be used in the materials design of future solar cells.

## Figures and Tables

**Table 1:** The average chemical composition of the annealed Cu_2_S–ZnS–SnS_2_ thin film samples.

	P1	P2	P3	P4	P5	P6	P7	P8	P9	CZTS
p_Cu_ (at. %) $\frac{\Delta p_{Cu}}{p_{Cu}^{CZTS}}\;\left(\%\right)$	20 −20	30 20	37 48	23 −8	27 8	30 20	22 −12	24 −4	29 16	25
p_Zn_ (at. %) $\frac{\Delta p_{Zn}}{p_{Zn}^{CZTS}}\;\left(\%\right)$	6 −52	7 −44	7 −44	10 −20	10 −20	9 −28	14 12	16 28	15 20	12.5
p_Sn_ (at. %) $\frac{\Delta p_{Sn}}{p_{Sn}^{CZTS}}\;\left(\%\right)$	24 92	17 36	12 −4	18 44	15 20	11 −14	16 28	12 −4	10 −20	12.5
p_S_ (at. %) $\frac{\Delta p_{S}}{p_{S}^{CZTS}}\;\left(\%\right)$	50 0	46 −8	44 −12	49 −2	48 −4	50 0	48 −4	48 −4	46 −8	50
$\frac{\Delta p_{S}}{p_{S}^{ideal}}\;\left(\%\right)$	−20	−18	−12	−15	−11	9	−17	−6	−6	0

**Table 2:** The percent of crystalline phases in each sample (*p_i_*—as-deposited; *p_a_*—annealed) and the ratio (*r* = *p_a_*/*p_i_*).

	*p_i_* (%)	*p_a_* (%)	*r*		*p_i_* (%)	*p_a_* (%)	*r*		*p_i_* (%)	*p_a_* (%)	*r*
P1	17	72	4.24	P2	33	79	2.40	P3	73	83	1.14
P4	32	55	1.72	P5	64	95	1.48	P6	52	95	1.83
P7	39	63	1.62	P8	82	82	1.00	P9	78	91	1.17

**Table 3:** The majority and minority phases after the sulfurization annealing of the Cu_2_S–ZnS–SnS_2_ samples. The PDF file number, the lattice system, and the space group were indicated for each crystalline phase. In addition, the sample chemical composition (*Cc*), the area of the peak at 2θ ~ 28.540° (*A*) and the average size of the crystallites of the majority phase (*d*) computed from this peak are given.

	*Cc* (%)	*A*	*d* (nm)	Crystalline Phases from GIXRD
P1	Cu: 20	420	17	65%: Cu_2.7_SnS_4_, tetragonal, I-42m (121), PDF 04-022-4944 7%: SnS_2_: hexagonal, P-3m1 (164), PDF 00-023-0677 28%: amorphous phase
	Zn: 6			
	Sn: 24			
	S: 50			
P2	Cu: 30	800	11	67%: Cu_3_SnS_3.6_, PDF 04-020-3908, tetragonal, I-42m (121) 12%: SnS_2_: PDF 00-023-0677, hexagonal, P-3m1 (164) 21%: amorphous phase
	Zn: 7			
	Sn: 17			
	S: 46			
P3	Cu: 37	1070	39	66%: Cu_2.7_SnS_4_, PDF 04-022-4944, tetragonal, I-42m (121) 17%: Cu_9_S_5_, PDF 00-047-1748, rhombohedral, R-3m (166) 17%: amorphous phase
	Zn: 7			
	Sn: 12			
	S: 44			
P4	Cu: 23	250	8	55%: Cu_3_SnS_3.6_, PDF 04-020-3908, tetragonal, I-42m (121) 45%: amorphous phase
	Zn: 10			
	Sn: 18			
	S: 49			
P5	Cu: 27	1950	37	93%: Cu_2.7_SnS_4_, PDF 04-022-4944, tetragonal, I-42m (121) 2% CuS: PDF 03-065-3556, hexagonal, P63/mmc (194) 5%: amorphous phase
	Zn: 10			
	Sn: 15			
	S: 48			
P6	Cu: 30	1190	35	74%: Cu_2.665_Sn_1.335_S_4_, PDF 04-009-7947, tetragonal, I-42m (121) 21%: CuS, PDF 00-006-0464, hexagonal, P63/mmc (194) 5%: amorphous phase
	Zn: 9			
	Sn: 11			
	S: 50			
P7	Cu: 22	780	12	63%: Cu_2.7_SnS_4_, PDF 04-022-4944, tetragonal, I-42m (121) 37%: amorphous phase
	Zn: 14			
	Sn: 16			
	S: 48			
P8	Cu: 24	630	52	45%: Cu_2.665_Sn_1.335_S_4_, PDF 04-009-7947, tetragonal, I-42m (121) 37%: CuS, PDF 03-065-3556, hexagonal, P63/mmc (194) 18%: amorphous phase
	Zn: 16			
	Sn: 12			
	S: 48			
P9	Cu: 29	2032	40	69%: Cu_2.665_Sn_1.335_S_4_, PDF 04-009-7947, tetragonal, I-42m (121) 19%: ZnS, PDF 01-074-4994, hexagonal, P-3m1 (156) 3%: Cu_1.96_S, PDF 04-007-1751, tetragonal, P43212 (96) 9%: amorphous phase
	Zn: 15			
	Sn: 10			
	S: 46			

**Table 4:** The wavenumber (in cm^−1^) of the Raman peaks, from the experimental Raman spectra reported in the literature for the polycrystalline phases, are given in the first column. The XRD 2θ positions of the most intense peaks, for these phases, and the Raman excitation wavelength are also indicated. The bolded values denote the most intense peaks, while the underlined values are for broad peaks.

Compound	Structure	XRD, Cu K_α_	Raman			
		2θ (°), (hkl)	λ_excitation_ (nm)	Peaks (cm^−1^)		
Cu_2_ZnSnS_4_	Tetragonal, I-42m	**28.484, (112)** 47.412, (204) 56.143, (312)	**514.5** [22]	285 **336** 362		
	Tetragonal, I-4	**28.473, (112)** 47.350, (204)/(220) 56.198, (116)/(132)	632.8 [23,24]	262.7 287.1 302.1	315.9 331.9 **337.5**	366.6 374.4
			514.5 [7,19]	252 272 287	331 **337**	347 353
Cu_0.5_Zn_0.25_Sn_0.5_S	Cubic, F-43m [25]	**28.525, (111)** 47.446, (220) 56.296, (311)	-	-		
Cu_2.7_SnS_4_	Tetragonal, I-42m	**28.583, (112)** 47.585, (204) 56.347, (312)	-	-		
Cu_2_SnS_3_	Tetragonal, I-42m	**28.566, (112)** 47.507, (204)/(220) 56.337, (116)/(312)	488 [26]	297 **336–337** 351		
	Cubic, F-43m	**28.470, (111)** 47.350, (220) 56.180, (311)	488 [26,27]	267 **303** 355		
ZnS [28]	Cubic, F-43m	**28.582, (111)** 47.555, (220) 56.337, (311)	514.5	275 **350**		
